# Perceptions on preeclampsia and eclampsia among senior, older women, in rural Southwestern Uganda

**DOI:** 10.29392/001c.19464

**Published:** 2021-03-03

**Authors:** Harriet Nabulo, Gad Ruzaaza, Francis Mugabi, Francis Bajunirwe

**Affiliations:** 1 Department of Nursing, Mbarara University of Science and Technology, Department of Nursing, Mbarara, Uganda,; 2 Department of Community Health, Mbarara University of Science and Technology, Mbarara, Uganda,; 3 Department of Community Health, Mbarara Regional Referral Hospital, Mbarara, Uganda

**Keywords:** uganda, rural, eclampsia, preeclampsia, perception, misconceptions, older, senior women

## Abstract

**Background:**

Eclampsia is among the leading causes of maternal mortality. It is a serious hypertensive complication of pregnancy and increases the risk of cardiovascular disease in later life. Pregnancy-related hypertension complications predispose to chronic hypertension and premature heart attacks. A significant proportion of women with preeclampsia/eclampsia does not reach the formal healthcare system or arrive too late because of certain traditional or cultural beliefs about the condition. The older, senior women in the community are knowledgeable and play a significant role in decision making regarding where mothers should seek maternal health care. Therefore, the purpose of this study was to explore the perceptions of older and senior women regarding the manifestation of, risk factors and possible causes of preeclampsia/eclampsia.

**Methods:**

We conducted a qualitative study in rural Southwestern Uganda. The key informants were senior, older women including community elders, village health team members and traditional birth attendants who were believed to hold local knowledge and influence on birth and delivery. We purposively selected key informants and data were collected till we reached saturation point. We analyzed data using an inductive thematic approach to identify themes.

**Results:**

We interviewed 20 key informants with four themes identified. The ‘*causes*’ theme emerged from the subthemes of confusion with other conditions, spiritual beliefs and high blood pressure. The ‘*risk factors*’ theme emerged from the subthemes of oedema-related illnesses, poverty-induced malnutrition, and strained relationships. The ‘*remedies*’ theme emerged from the consistent mention of traditional herbal treatment, seek medical help, spiritual healing, emotional healing and corrective nutrition as potential solutions. The theme ‘*effects of preeclampsia/eclampsia*’ emerged from the mention of pregnancy complications like premature delivery, child loss, operative delivery like caesarian section delivery as well as death. There was no identifiable local name from the interviews. Women carried several myths regarding the cause and these included little blood, witchcraft, ghost attacks and stress from strained relationships including marital tension. Women were generally aware of the outcomes of eclampsia, mainly that it kills.

**Conclusions:**

Eclampsia is associated with significant myths and misconceptions in this rural community. We recommend interventions to increase awareness and dispel these myths and misconceptions, increase access to antenatal preeclampsia surveillance, and facilitate timely referral for basic maternity care as means for early detection and management of preeclampsia.

Preeclampsia is a pregnancy complication characterized by high blood pressure and signs of damage to organ systems most often the liver and kidneys. Eclampsia is a severe complication of preeclampsia where high blood pressure results in seizures during pregnancy and is life threatening. Both preeclampsia and eclampsia are pregnancy-specific conditions resulting in hypertension and multi-organ dysfunction.^1,2^ Hypertension and other forms of cardiovascular disease (CVD) are a leading cause of death and in sub Saharan Africa, women are more likely to die of heart disease compared to men.^3,4^ Overall, incidence of heart disease and other non-communicable diseases are on the rise in resource limited settings.^5^

Prior history of preeclampsia or eclampsia increases risk of cardiovascular disease^2,6,7^ and diabetes^8^ later in life. Preeclampsia is associated with a 4-fold increase in future incidence of heart failure and a 2-fold increase in the risk of coronary heart disease, stroke, and death from a cardiovascular disease - related event.^1,2,9–12^ Hypertensive disorders complicate 5% to 10% of pregnancies.^13^

Uganda’s maternal mortality ratio is estimated at 368 deaths per 100,000 live births, and approximately 15 pregnant women dying every day due to direct causes like hemorrhage and hypertensive disorders.^14^ Almost 15% of maternal mortality in southwestern Uganda is attributable to hypertensive disorders in pregnancy mainly eclampsia from women admitted in critical condition.^15,16^ Preeclampsia and eclampsia are among the top three causes of maternal mortality worldwide^17^ and the incidence remains high in resource limited settings, although it has reduced in resource rich countries.^18^ Between 2003 and 2009, hemorrhage, hypertensive disorders, and sepsis were responsible for more than half of maternal deaths worldwide with hypertensive disorders contributing 14% to direct maternal mortality.^19^ In sub-Saharan Africa, about one third of the mothers with eclampsia will experience a still-birth and eclampsia is responsible for almost 20% of maternal mortality and disability.^20^ Maternal mortality may be higher in rural areas due to difficulty in access to services as shown in a recent study in Southwestern Uganda where most of the maternal deaths were related to late referrals.^15^ Rural women often experience delays to make a decision to seek care, delay to reach place of care and delay in receiving appropriate and adequate care.^21,22^ The decision to take up referral and deliver at a higher level facility may be influenced by community perceptions regarding the conditions for which the patient is referred.

Perceptions towards a health condition are influenced by several facets such as culture, personal beliefs, experiences and knowledge.^23^ Studies conducted in Asia and West Africa suggest there is a variety of community perceptions that may be barriers for women with pre-eclampsia to seek care and eventually deliver at a health facility.^24–26^ The clinical presentation of preeclampsia and eclampsia may not be well understood by some of the communities, and is often confused with other conditions; some communities believe that local home remedies may cure them.^24^ Some communities associate preeclampsia/eclampsia with witchcraft.^27^ These alternative explanations may lead to late care seeking for mothers with preeclampsia and this is often further compounded by weaknesses in the existing health care system that prevail in low resource settings.^28^

Older and senior women in the communities and families play a significant role and decision on where women will deliver.^29,29,29,30^ The perceptions they carry about certain health conditions may influence pregnant women’s decision to seek care or deliver in health facilities.^31^ There is limited data on studies to explore the perceptions of older, senior women on preeclampsia/eclampsia in resource limited settings with poor access to health care. Therefore, the purpose of this study was to explore the perceptions of older and senior women regarding manifestation, risk factors and possible causes of preeclampsia/eclampsia in rural southwestern Uganda. We hypothesized that these perceptions may be driven by cultural beliefs, myths and misconceptions which may contribute to staying away or delay in seeking care by women with preeclampsia in rural South-western Uganda.

## METHODS

### STUDY DESIGN

This was a cross-sectional qualitative study conducted using key informant interviews. We interviewed older and senior women and documented their perceptions on preeclampsia/eclampsia.

### STUDY SETTING

The study was conducted in Kabuyanda subcounty, in Isingiro district of southwestern Uganda, a rural remote location. The terrain in this district is very hilly, making transportation very challenging. The village is the smallest administrative unit in the local government of Uganda. A village usually consists of between 50 and 70 households and may be home to between 250 and 1,000 people. A collection of six or seven villages makes a parish. About five to seven parishes make up a sub-county and three or four sub-counties make county.

**Figure 1.**
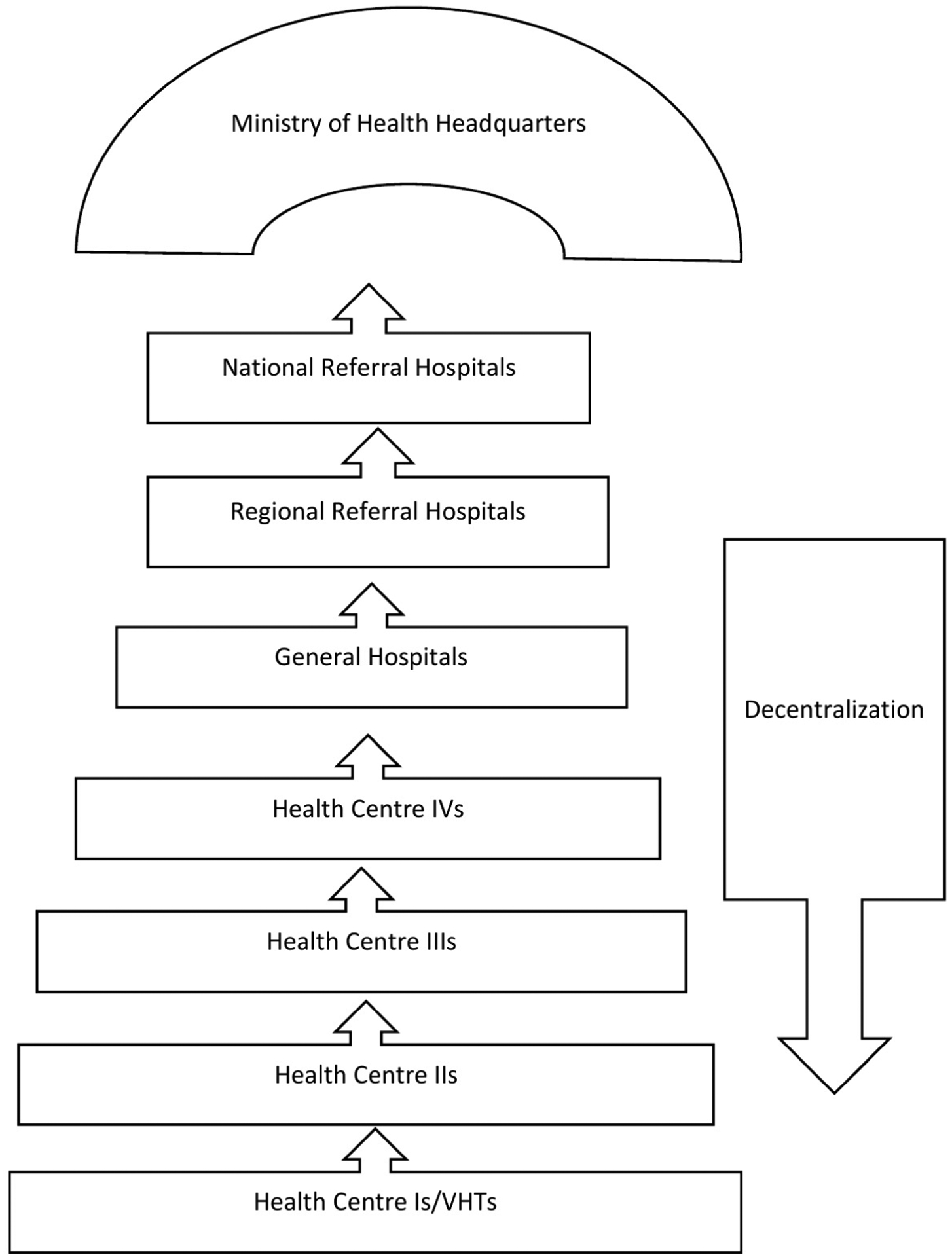
Health care system in Uganda.

The health care system is decentralized. At village level, the village health team (VHT) serves as a satellite site, with no definite physical structure for services such as outreach for immunization and health education. HC II offers preventive, promotive, outpatient curative health services, and outreach care. HC III offers preventive, promotive, outpatient curative, maternity and inpatient health services and laboratory services. HC IV offers preventive, promotive and outpatient curative, maternity, inpatient health services, emergency surgery, blood transfusion, laboratory services and referral services. Figure 1 represents structuring of the health care system in Uganda.

The sub-county is served by two health centres (both HC IIs), namely Rwakakwenda and Rwamwijuka. The two health facilities do not offer antenatal care services. Most women in this sub-county are referred to Kabuyanda health centre (HC IV) which is relatively far away, approximately 30-minute to 1-hour journey on a motorcycle taxi. Kabuyanda health centre refers cases they are not able to handle, to Mbarara Regional Referral Hospital (MRRH), a journey that takes well over 1 hour. By the time women from this community arrive at the biggest regional referral hospital for basic and comprehensive emergency services, they are often very ill with complications.

HC IVs to national referral hospitals constitute the tertiary/level III levels of care. HC IIIs and IIs are the secondary level/level II and HC Is are the primary level care/level I. In Uganda’s health care delivery system, a HC II does not provide antenatal care (ANC) and birthing services. Pregnant women in our study setting may be at a relatively higher risk of developing undiagnosed pre-eclampsia due to difficulty in accessing antenatal blood pressure check- ups. This study area was chosen because Mbarara Regional referral hospital receives a significant proportion of mothers from this region, often referred very late with eclampsia.

### SAMPLING STRATEGY AND STUDY POPULATION

We used purposive sampling to recruit the study participants. We explained our inclusion criteria to the parish mobilisers who moved with us home to home of our potential participants or mobilized some to come to the parish headquarters for interview. We were two teams. Participants who consented to participation were interviewed at the parish headquarters or some in their homes. Data were collected over a 2-week period in April 2018.

The key informants were senior and older women aged between 45 and 79 respectively. These were chosen because they are believed to be the custodians of traditional beliefs and practices that may affect health-seeking behavior. The key informants (KIs) were recruited as they met the inclusion criteria during the data collection period. They were of higher parity; their experience with numerous births was thought to increase their possible encounter with complications of pregnancy, either for themselves or their acquaintances. They consisted of traditional birth attendants (TBAs) and VHT members. TBAs have learned to deliver babies, usually from an older woman and run maternity services for the community from their homes. VHT members are lay persons usually three or four per village, tasked with overseeing and mobilizing for health-related activities in the village, risk identification and referral; so would make an important key informant resource. The study was conducted in the five parishes of the sub-county. The study teams listened to the audios at the end of a day’s work. After 20 key informant interviews, we noted that no new information was being generated, and at this point we considered that data saturation had been reached.

### DATA COLLECTION TOOLS

We designed an interview guide to collect data. The interview guide had sections on socio- demographic characteristics of the participants. We asked participants their age, occupation and level of education. We designed open–ended questions describing preeclampsia and eclampsia as they present in pregnancy and asked participants to mention the perceived local name for disease, what they thought was the cause and risk factors and how the condition should be managed. Participants were asked to name a disease that presents with blurred vision, severe headache, right upper quadrant abdominal pain, fitting, suggested remedies and the short term and long-term effects of eclampsia.

We recruited three experienced research assistants (RAs) and trained them to administer the interview guide. One of the co-authors (HN) worked with the RAs during the training and conducted role plays with the tools to ensure there was clear understanding of the questions. The RAs were exposed to the study tools to get acquainted with them in the two-day training. We conducted pilot interviews with 4 older women in a contiguous parish, not the study parish, to test the tool in a similar setting to ensure the questions were valid. We made a few adjustments following the pilot.

### DATA COLLECTION AND ANALYSIS

The data collection was done by a team of four: three RAs and one of the co-authors (HN) and they formed two teams. Each team comprised 2 interviewers, who were all female and each one took turns at interviewing. We obtained an introductory letter from the Assistant District Health Officer (ADHO) and presented this to the parish mobilisers as our port of community entry. Parish mobilisers are volunteer community members who mobilize for health campaigns and activities in their locality such as mass mosquito net distribution, vitamin A supplementation and mass immunization campaigns. These mobilizers assisted in identification of the eligible participants and the RAs recruited them into the study. All the participants approached accepted to be interviewed. The interviews were conducted in the local language, *Runyankore-rukiga*, and in private rooms at the parish headquarters and some in their homes. Data collection was stopped when the point of saturation was reached. Interviews lasted between 40–60 minutes and were audio recorded. At the end of each day, two co-authors (HN and GR) listened to the audios together with the rest of the interviewing teams.

The transcripts were translated into English by a native *Runyankore-rukiga* speaker. Two co-authors (HN and FM) are fluent in the local language and listened to all the audios to ensure they corresponded with the transcriptions. The corresponding author read and re-read the transcripts to ensure they were complete. We used an inductive thematic approach to the data analysis. Data analysis was done in three phases: preparation, organization and results’ reporting as described elsewhere.^32–34^ The teams read through and examined the data, identified coding units, analyzed data by applying the coding units, made a tally of the number of times a coding unit had appeared.^35^ The unit of analysis was a key informant interview. The questions in the interview guide also guided in the naming of the themes that we came up with. Data were coded by two teams separately (HN and GR, FM and FB), identifying sub-themes and themes. The teams reviewed the themes to reconcile any differences. We used NVivo software version 12 to complete analysis.

### ETHICS CONSIDERATIONS

We obtained ethical approval from the Mbarara University of Science and Technology Research Ethics Committee and the final approval was issued by the Uganda National Council for Science and Technology. Administrative clearance was granted by the Assistant District Health Officer (ADHO) of Isingiro district. The ADHO provided introductory letters to the local authorities permitting data collection in the villages of the county. We explained the details of the study procedures to the participants, once they understood and agreed to participate, we obtained written informed consent. Participants who could not write, were asked to append their right thumb prints in place of signatures. The identity of the participants was kept anonymous. Data were accessible to only the study team.

## RESULTS

We enrolled 20 participants and reached data saturation. The largest proportion were aged between 45 and 49 years, were married, belonged to the Bakiga tribe. Details of the socio-demographic characteristics are shown in Table 1. Most of the women were either members of the village health team or traditional birth attendants. The largest proportion of them did not have a formal education.

### IDENTIFIED THEMES

The questions we asked the women guided us in the development of themes. Four themes were identified from our analysis namely; ‘causes’, ‘risk factors’, ‘remedies’, and ‘effects of preeclampsia/eclampsia.’ These are presented in Table 2. The ‘*causes*’ theme was coined from the subthemes of confusion with other conditions, spiritual beliefs and high blood pressure. The ‘*risk factors*’ theme was made up of the sub-themes of oedema-related illnesses, poverty-induced malnutrition, and strained relationships. The ‘*remedies*’ theme resulted from the consistent mention of traditional herbal treatment, corrective nutrition, seek medical help, spiritual remedies, and emotional/psychological healing as potential solutions cited by the majority. The ‘*effects*’ theme was devised from the mention of pregnancy complications like premature delivery, child loss, operative delivery like caesarian section delivery and death as potential consequences.

## CAUSES

### CONFUSION WITH OTHER CONDITIONS

Participants thought that the convulsions that occur in this disease can be caused by “*omuraramo* or *ensimbo”* literally translated as “meningitis or epilepsy” respectively in the local Runyankore-rukiga dialect. *Ensimbo*, translated as ‘epilepsy’ was confused with pre-eclampsia by some of the women. Participants said that fits/convulsions in pregnancy are due to epilepsy especially if it has been pre-existing before the woman got pregnant. This is probably because epilepsy may mimic eclampsia by fitting owing to occurrence of fits.

‘Fitting in pregnancy is caused by [ensimbo]. It’s a bad omen to suffer from epilepsy. When someone is convulsing because of epilepsy, we run away from them because should they pass flatus when one is near them, he catches it too. It’s terrible, you don’t want your relative to have epilepsy of all diseases.’

48-year-old, peasant, mother of 6.

‘Those symptoms could be caused by omuraramo [meningitis] and ensimbo [epilepsy] but now you see the confusion is that even men can have it and can fit.’

45-year-old TBA, mother of 3.

### SPIRITUAL BELIEFS

There was a common belief in spirits and ghosts. Some respondents thought the fits were linked to a ghost attack from recent demise of a close relative especially if the pregnant woman was either not at peace or was liked too much by the deceased.

‘Some relatives don’t die completely. If at the time of demise, you had a misunderstanding or they loved you too much and are not resting in peace, they may strangle you so that you die too.’

73-year old widow, mother of 7.

Several respondents believe the illness may be a result of witchcraft especially from persons that do not wish the pregnant woman well.

“Not everyone is happy for you when you get pregnant, for some reason, someone can bewitch you and you fit, a co-wife for example could wish you dead. When you are pregnant, she is already imagining that your child will compete for property with her children”

45-year-old VHT, mother of 5.

## RISK FACTORS

### POOR FEEDING

The respondents associated the oedema or body swelling seen in preeclampsia and eclampsia patients with having anemia or ‘little blood’ in the pregnant woman’s body. Respondents mentioned that a poor diet dominated by *matooke* [steamed bananas] and no beans or green vegetables or even millet porridge was to blame for the ‘little blood’ in women.

‘When you eat poorly, especially when you can’t find foods that give blood, then your whole body will swell. In our days, our diet used to be dominated by green vegetables, bean soup and millet porridge; rarely did women swell feet because of little blood. Young women these days shun these foods; that’s why they swell the body due to little blood”

57years TBA, mother of 5.

Respondents mentioned that poor feeding resulting from a pregnant woman not being able to find foods rich in nutrients such as iron, not having enough to eat in general due to lack of money to buy the necessary food stuffs was a cause for ‘little blood’ and body swelling in pregnant women.

‘I think the person is weak in this case and does not have enough blood because of poor feeding, food insecurity; you know village life where people don’t have money to buy foods rich in iron, like the health worker tells us to eat meat and fish, so this may be the cause for the swelling in pregnant women, then the stress associated with this lack will bring about headache. As you know, our poor way of living in the village may have brought about this,’

45-year-old VHT member, mother of 6.

This was echoed by other participants who thought not taking the right foods is solely responsible for body swellings in pregnant women.

‘……. the woman was not having a balanced diet. At times the pregnancy restricts someone and they have no appetite, the cravings also. A woman may end up eating matooke(bananas) with only salt, they don’t want beans or other times they depend on only drinking water. Such a person may lack blood and end up getting swollen feet or even the whole body.’

56 years, women’s leader, mother of 5.

‘The challenge is that some pregnant women are not feeding well or have other diseases that are left untreated and can swell up or even fit and so they will blame pregnancy when it isn’t the case’

45-year-old VHT, mother of 4.

### STRAINED RELATIONSHIPS

Some participants thought that life stressors like strained relationships, abusive spouses can cause stress resulting in a headache. They mentioned that some women are in polygamous relationships with nagging co-wives. Coupled with poverty, they are unable to have enough nutritious foods such as those rich in iron. They believed that all these factors may cause a rise in one’s blood pressure especially when pregnant.

‘For us we always think that she has a lot of thoughts like when the woman is unstable and unhappy at home, like the man doesn’t bring for her home necessities and when she encounters such problems, they can cause a headache and ‘puresha’ [high blood pressure]. Some are in polygamous relationships that cause them additional tension as if the poverty they are experiencing is not stressor enough.’,

48-year-old, Peasant, mother of 6,

### OEDEMA-RELATED ILLNESSES

Multiple pregnancies are associated with a certain level of prestige but seemed to be associated with complications such as body swelling and anemia.

‘They are not many though I usually see some women with swollen hands, face and legs and I always think it is because one is expecting twins and at times, we joke about it. Having twins is a blessing but going through multiple pregnancy is no easy task’

45 -year old, VHT member, mother of 6.

Participants believed that body swelling was predictive of the size of the baby the woman was carrying, and some respondents seemed to suggest that the more the swelling, the bigger the baby would be when born.

‘We think that a woman swelling during pregnancy means that she has a big baby who is demanding a lot of blood from the woman, hence the body swelling.’

56-year-old, women’s leader, mother of 5.

### HIGH BLOOD PRESSURE

Participants especially the VHT members seemed to correctly relate having high blood pressure with symptoms of pre-eclampsia and eclampsia as evidenced in the narrative from a VHT member. This is probably because VHT members in Uganda have received some training on identification of obstetric danger signs.

‘The signs that she has, the woman who has [high blood] pressure, she has severe headache, she tells you that the heart pumps a lot like it is about to fly out of the chest, the feet swell, she can fit sometimes; these are the only signs I know. I don’t do much with such a woman, I just give her a referral letter to hospital immediately. There is nothing more I can do really because am not empowered to help her’.

45 year, VHT coordinator, mother of 4.

## REMEDIES

### HERBAL TREATMENT

Participants had different views on how a woman with preeclampsia/eclampsia should be managed. The recommendations ranged from herbal, medical treatment to referral to the formal health care facilities.

Some of the participants said that when getting medical treatment from the hospital is difficult, they resort to some herbal concoctions. The respondents believed that these had the ability to raise their blood levels and therefore the swelling would reduce.

‘The woman goes to a health facility and gets some tablets but these may not be very helpful most of the time. At times they go to Kabuyanda [county level] HC and are not helped so they are advised to seek further medical treatment in a hospital. But they do not go because they have no money. So, they resort to using herbs. …they can use a red herbal concoction and sometimes beet root that increases their blood levels’

a 45-year-old VHT member, mother of 6, with 2 abortions.

### SPIRITUAL REMEDIES

Some participants thought convulsions in a pregnant woman are linked to a ghost attack from recent loss of a close relative. They mentioned that the traditional healer would appease the dead by performing certain rituals.

‘The services of a traditional healer should be sought to appease the dead. I don’t know what the traditional healer does, but I know that he should be performing some rituals to appease the dead’

73-year old widow, retired TBA, mother of 7

### CORRECTIVE NUTRITION

Participants alluded to a balanced as being essential; their understanding of a balanced diet meant eating beans, green vegetables, sweet potatoes, and millet porridge to correct the anemia. They believe that this type of diet will correct the dizziness and body swelling and prevent the convulsions as well.

‘At times the pregnancy is very demanding on the woman to carry but also this is due lack of enough blood. When they are swollen up, they are encouraged to feed well so that they can get enough blood like feeding on liver can restore the blood levels and the body swelling will slowly go away. When the body is swollen and when they visit the hospital, they are advised on how to feed; on foods like green vegetables and millet porridge. Enough blood prevents them from fitting as well.’

65 years VHT, mother of 6.

### EMOTIONAL/PSYCHOLOGICAL HEALING

Some participants believed that symptoms related to eclampsia were because the woman was being mistreated by her husband. They believed that if the husband treated her better and took good care of her, these symptoms would resolve or would not appear in the first place.

‘Loving each other and getting due attention from husband when a woman feels any pain, helps a lot. Also, a husband who treats his wife well saves her stress that can cause her [high blood] pressure.’

65 years, VHT member, mother of 6.

Participants believed in emotional healing as a remedy for eclampsia. They thought prayers and counselling can help to calm the stressed woman.

‘Seeing a counselor can help her to find peace since they have troubled lives. She can join a prayer group and pray for peace in her home to save her the stresses of daily life.’

60-year-old farmer, mother of 7.

### SEEK MEDICAL HELP

Although some participants were proponents of local herbal remedies, some strongly discouraged them and recommended visiting the health facility.

‘Long time ago herbs used to help in every illness of our forefathers but nowadays they tell you they take them but the herbs are of no help. So, they are better off going to hospital for treatment because herbs these days are no longer potent.’

45-year-old VHT, mother of 4.

## EFFECTS OF PREECLAMPSIA AND ECLAMPSIA

Participants were aware of the potential consequences of the symptoms of eclampsia. They mentioned that the condition could lead to death of the baby in utero or even the mother. These potential adverse outcomes were mentioned by the majority of participants.

‘The person may die if not referred to hospital. If the woman does not die, her baby will die in the womb. How can the baby survive with a mother that has no blood?’

45 year, VHT coordinator, mother of 4.

Most of the participants linked the signs and symptoms of preeclampsia to little blood that causes body swelling. They said that such women tend to have premature deliveries.

‘…Maybe the baby can become very weak or some women get swollen legs, little blood and this can sometimes lead to premature births. That is how we see them.’

60-year old farmer, mother of 7.

### OPERATIVE DELIVERY

Participants thought that women with symptoms of preeclampsia/eclampsia become very weak; fail to push the babies necessitating surgical operations to deliver their babies.

‘…No, it’s not that they get all that well, they remain weak and at the time of giving birth they may still be weak, they may fail to push the baby and are delivered by caesarian section.’

45-year-old VHT member, mother of 6.

### DEATH

Participants acknowledged that preeclampsia/eclampsia is a serious and life-threatening illness. They agreed that if not attended to, the disease had some grave consequences including the potential to cause death.

‘I think such a person should get help from a health facility but because we are in the village at times when a woman has such a condition and you tell her to go to hospital, their husbands don’t care, in such a situation, the woman remains at home in that poor state of health and at times she dies.’

57 year old TBA, mother of 7.

## DISCUSSION

Our data from rural southwestern Uganda shows that majority of the women understood that eclampsia was related to high blood pressure and is also potentially fatal. Although, a distinct local name did not emerge, participants related the condition to other medical conditions namely epilepsy and meningitis due to a shared symptom of convulsions. The women described various factors that they believe may be causes and remedies for the condition, most of which had no direct relation with eclampsia.

The descriptions women gave were made in reference to other diseases namely; meningitis and epilepsy. Anecdotal evidence from midwives practicing in central and other places in southwestern Uganda where the *Bakiga*, the predominant tribe in our study area live, refer to eclampsia as ‘*amakilo’* and *‘amakiro’* in central and southwestern Uganda respectively. We were surprised that a clear local name for eclampsia did not emerge for this community that is also largely comprised of the same *Bakiga* tribe. It is not clear whether migration could have caused the loss of the disease name. A local name attached to preeclampsia and eclampsia with an identifiable set of symptoms is desirable as this gives the condition local identity and enhances uptake of interventions to manage it.

Data from a study in rural Nigeria^25^ and Pakistan^36^ support our finding that a local name may not necessarily exist. In the Nigerian study done among the *Yoruba* tribe, respondents described the condition as the epilepsy of pregnancy, similar to what participants in our study did.

Our data show that when women were asked about causes and risk factors for eclampsia, they revealed a high level of misconception regarding the condition. The women suggested several potential causes for eclampsia and these included; anemia or having little blood, carrying a very big baby, poor feeding, witchcraft and even marital stress. Misconceptions are common in sub Saharan Africa. In a qualitative study in Nigeria^25^, women also suggested that marital conflict, abusive husbands and strained relationships were responsible for eclampsia.

In a similar study in Mozambique designed to examine community knowledge about preeclampsia, women also believed that it is caused by stress, worry and mistreatment from in-laws.^27^ In this Mozambican study, extreme suggestions such as snakes living inside the woman’s body were fronted as possible explanations. Witchcraft was mentioned as a possible cause in our study and the Mozambican one. Despite these prevailing misconceptions, some participants correctly associated eclampsia with high blood pressure.

There were several misconceptions regarding the remedies for preeclampsia. The local remedy most participants easily turned to was herbal remedies. Herbal remedies are common throughout sub Saharan Africa as a first mode of treatment. The concern is these remedies provide false hope and may cause delay in seeking treatment. One study in Nigeria^37^ found that use of herbs was associated with severe eclampsia and preeclampsia. Women also suggested prayer as a potential remedy. While there are strong religious beliefs in sub Saharan Africa, these may contribute to the delay in seeking care or the first delay.^21,38^

Respondents were aware of the dangers of eclampsia and expressed fear for maternal deaths as the ultimate consequence of eclampsia. This awareness of the dangers of eclampsia provides a window of opportunity for interventions to target even the wider challenge of the misconceptions. The health belief model has been used to explain poor health care seeking behaviors in Nigeria owing to misconceptions.^39^ The perceived susceptibility to still birth and death among mothers with eclampsia as reported by these senior influential women in our study and the acknowledgement by some that medical attention should be sought could provide a valid basis for interventions. Community health workers provide a potential first line of intervention as they have been shown to have sufficient knowledge and ability to identify women with preeclampsia and administer initial treatment.^40–42^ Future programs will need to develop interventions that focus on demystifying the prevalent myths and promote a more scientific understanding of the condition and eventually this knowledge could serve as cues for action.

Our study has important strengths. Although several studies have been conducted on this subject, our study focuses on the senior and older women who play a significant role in the health care seeking behavior of pregnant women. Second, our study is community-based in a rural population. The data collected will inform interventions to improve outcomes of women with eclampsia. These participants are the custodians of local knowledge and have the power to influence belief and practice of younger women and eventually their health seeking behavior. Third, our study reveals some unique myths and misconceptions that should be explored in other communities as well. There are some limitations to our study. We used only key informant interviews to collect data. A combination of focus group discussions (FGDs) and key informant interviews (KIIs) could have enabled us to obtain richer data. However, we ensured that we obtained as much information as we could from the KIIs. Secondly, we were only able to enroll a handful of participants above 60 years of age. In the initial stages of the interviews, we gave the participants a list of the symptoms and signs of preeclampsia/eclampsia and asked them to state which disease could present this way. This description of the disease could have pre-conditioned their responses. However, we asked a similar question in a paraphrased, open-ended manner to elicit their open view. Additionally, efforts to enroll more older women in this community were futile as there were only a few.

In conclusion, our study in rural southwestern Uganda has shown that women were generally aware of the potential danger of pre-eclampsia. However, there is no identifiable local name for preeclampsia that can be easily tagged to the condition. The community holds a lot of myths that surround the possible causes and risk factors for preeclampsia and these may negatively influence health care seeking behavior. There is a great need to raise awareness about preeclampsia and dispel the myths surrounding it, increase access to antenatal preeclampsia surveillance, and facilitate timely referral for basic maternity care as means for early detection and management of preeclampsia.

## Figures and Tables

**Table 1.** Socio-demographic characteristics of the interviewees N=20

Variable	n=20
*Age*	
65 and over	2
60–64	4
55–59	4
50–54	3
45–49	7
*Marital status*	
Married	18
Widow	2
*Tribe*	
Mukiga	19
Nyankore	1
*Parity*	
1–5	9
6–10	9
>10	2
*Education level*	
None	12
Lower primary	6
Lower secondary	2
*Occupation*	
Traditional Birth Attendant	8
Village Health Team	9
Peasant farmer	2
Chairperson for women committee	1

* lower primary consists of 4 years of education and 3 years of upper primary education; lower secondary school has 4 years of education.

**Table 2.** Identified themes on perceptions of pre-eclampsia and eclampsia

Codes	Sub-themes	Themes
*Omuraramo* and *ensimbo*	Confusion with other conditions	Causes
Ghost attack	High blood pressure	
Witchcraft	Spiritual beliefs	
*Puresha* (high blood pressure)		
Marital tension and stress from nagging co-wives	Strained relationships.	Risk factors
A disease of rich people	Oedema-related conditions	
Poverty	Over-fed (obese) women	
Alcohol intake	Malnutrition due to poverty	
‘Little’ blood		
Lack of craved foods		
Big baby		
Poor feeding		
Multiple pregnancy		
Herbal treatment	Traditional medicine	Remedies
Prayers and Counseling	Corrective nutrition	
Corrective nutrition	Seek medical help	
Appease the dead	Spiritual remedies	
“Good treatment” by spouse	Emotional/psychological healing	
Go to hospital		
Premature delivery	Pregnancy complications	Effects of preeclampsia/eclampsia
Still birth	Child loss	
Caesarian section delivery	Operative delivery	
Death	Death	

## Data Availability

These are available from corresponding author upon reasonable request and with approval from the Research Ethics committee.
